# Subjective and Objective User Behavior Disparity: Towards Balanced Visual Design and Color Adjustment

**DOI:** 10.3390/s21248502

**Published:** 2021-12-20

**Authors:** Anna Lewandowska, Agnieszka Olejnik-Krugly, Jarosław Jankowski, Malwina Dziśko

**Affiliations:** Department of Computer Science and Information Technology, West Pomeranian University of Technology, Żołnierska 49, 71-210 Szczecin, Poland; aolejnik@wi.zut.edu.pl (A.O.-K.); jjankowski@wi.zut.edu.pl (J.J.); mdzisko@wi.zut.edu.pl (M.D.)

**Keywords:** website design, color contrast, WCAG, accessibility

## Abstract

Interactive environments create endless possibilities for the design of websites, games, online platforms, and mobile applications. Their visual aspects and functional characteristics influence the user experience. Depending on the project, the purpose of the environment can be oriented toward marketing targets, user experience, or accessibility. Often, these conflicting aspects should be integrated within a single project, and a search for trade-offs is needed. One of these conflicts involves a disparity in user behavior concerning declared preferences and real observed activity in terms of visual attention. Taking into account accessibility guidelines (WCAG) further complicates the problem. In our study, we focused on the analysis of color combinations and their contrast in terms of user-friendliness; visual intensity, which is important for attracting user attention; and recommendations from the Web Accessibility Guidelines (WCAG). We took up the challenge to reduce the disparity between user preferences and WCAG contrast, on one hand, and user natural behavior registered with an eye-tracker, on the other. However, we left the choice of what is more important—human conscious reaction or objective user behavior results—to the designer. The former corresponds to user-friendliness, while the latter, visual intensity, is consistent with marketing expectations. The results show that the ranking of visual objects characterized by different levels of contrast differs when considering the perspectives of user experience, commercial goals, and objective recording. We also propose an interactive tool with the possibility of assigning weights to each criterion to generate a ranking of objects.

## 1. Introduction

The digital design of websites, games, or other forms of online publishing, in addition to requiring valuable content and a friendly design to promote a positive user experience, requires the proper display of visual objects, including their colors and contrast. The connection of several factors and possibilities generates dilemmas related to interaction design, the use of persuasive elements, and impacts on user behaviors. From the perspective of marketing, the design is focused on user conversions and steering user behavior according to business goals [1,2]. Camgöz [3] researched the influence of color on customers’ perceptions and purchase decisions. The application of high color saturation promotes user excitement and increases color perception which, in turn, causes greater readiness for action and payment. Designers need to cope with phenomena like banner blindness [4,5] or habituation to attract user attention [6,7]. Intensive visual content and invasive techniques that break cognitive processes are often used; however, they lead to a negative user experience and may impact brand perception. Marketers use high contrast to attract user attention, which can be distracting during system usage and, as a result, lower the user experience.

**Figure 1:**
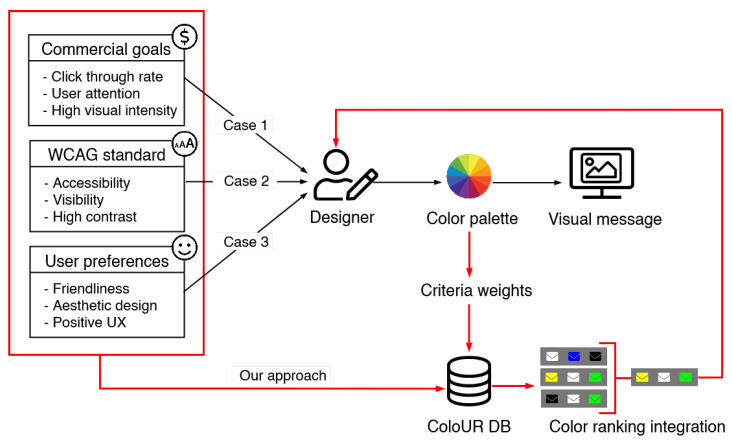
A designer must take numerous factors into account. On one hand, their work represents the implementation of their creative vision, while on the other hand, business requirements, standards, and technological limitations must be considered. The fulfillment of business targets is measured by the attainment of a high click-through rate (CTR) [11] through the use of visual design to attract the user’s attention, especially in the color domain (Case 1). Trends, as well as standards, such as the WCAG, impose visual design adjustments on different social groups by, e.g., limiting the color palette to colors with a high contrast factor (Case 2), whereas the user follows their preferences and expects a friendly and aesthetic visual message, which is simultaneously legible and comprehensible (Case 3). Thus, the search for a solution that balances user preferences, the attraction of user attention, and the fulfillment of WCAG standards takes place. The used dimensions are subjective data, objective measurements, and color contrast. Data collected during the experiment are stored in a knowledge database, called ColoUR DB (Color User Response Database). The designer decides which criteria are relevant for a given visual design and adjusts their weights appropriately. Based on the designer’s settings, a color ranking is generated. It is possible to obtain a color ranking for each separate criterion as well as an integrated one that covers all designers’ settings. The results were used to develop and implement a tool for designers, which we call The ColoUR Balance Tool.

From another perspective, the main goal of design should be to create a friendly environment with characteristics that are acceptable to a wide group of target users, including users with disabilities (see Figure 1). Common rules are delivered by the Web Accessibility Guidelines (WCAG) [8], which are recommended for content design. One of the aspects is based on contrast, which assures effortless reading of presented information: the greater the contrast, the easier it is to differentiate an object, photo, or foreground text from the background. A low contrast level hinders website usage, especially for users with lower sight sharpness (not only those with eye defects, but also the elderly) as well as those who are visually impaired. Therefore, the main purpose is to deliver comfort to users with disabilities and reduce barriers to usage. This has great importance, with 15% of the population having some kind of disability [9]. It is worth noting that cognitive, hearing, or visual disabilities may result in difficulties during the use of online system use [10].

However, Schmutz et al. emphasized that the WCAG are rarely implemented in practice, and about 95% websites do not meet these standards, as confirmed by earlier studies [12]. Various reasons for such a situation have been reported. including problems with achieving commercial targets, the possibility of designing better products without limits [13], legal factors [14], and implementation limits [15]. While the main assumptions and motivation behind the WCAG are clear, and the awareness of their existence is common, the lack of direct financial benefits and influence from the majority of users and business clients is another factor that decreases the use of the accessibility guidelines [16,17]. It has also been reported by earlier studies that websites following the WCAG can be perceived as boring and not visually attractive [18]. Limited usage of WCAG rules is also related to concerns about a potential usability drop for non-disabled users [19,20]. As a result of various concerns, Karreman et al., among others, emphasized that it is not easy to achieve a trade-off between targeting the design toward users with disabilities while still maintaining positive perceptions by non-disabled target groups with the same design [19]. However, recent studies showed that the WCAG also has the potential to be used to design websites with high usability for non-disabled users [12,21].

The final design should follow user preferences and abilities. Models of friendly color combinations are used in the design process to deliver designs with high readability and appropriate contrast, allowing effective communication with a user. Nowadays, designers have a multitude of tools at their disposal for picking colors and even whole design compositions or website templates. Basically, they can be divided into mathematical models and user-preference-based tools. The former allows the designer to choose the main color, and algorithms pick the remaining colors; examples of such tools are Adobe Color (color.adobe.com) and Paletton (paletton.com). The second group consists of tools based on preferences, trends, and artists’ visionary work; examples are Behance (behance.net), Dribbble (dribbble.com), and the Colour lovers tool (colourlovers.com). All of these solutions implement particular goals (business, user preferences), but none of them take all of those goals into account at the same time, trying to deliver solutions that apply business goals, meet user preferences, and fulfill the requirements of the WCAG standards.

Satisfying accessibility standards and user preferences create design dilemmas, and complexity grows with the addition of marketing goals and the need to attract user attention. Marketing goals may invade user experience and can conflict with accessibility rules. The designer must also consider that visual messages are directed to different recipient groups and will be different from each other. A commercial message encouraging purchases will have different goals than a social or informational message. However, social and economic changes, as well as social media, diminish the line between recipient groups, and visual messages travel through different channels and get to different groups; this often happens without the control or intention of the designer. More and more often, visual messages are designed such that they capture characteristics that are important to various groups, e.g., visual messages designed for disabled (temporarily and permanently) people will be understood by older people and their caretakers. This change in thinking can be observed by the increasingly muted approach to design—in terms of both service design and user experience [22].

Therefore, taking into account the above, our research goals were to examine how visual message characteristics in the color domain depend on the chosen goal: commercial goals, represented by the visual intensity, with attention measured automatically by eye-tracker (objective data); the fulfillment of the WCAG standards, and user preferences (subjective data). Could the observed disparity in objective data (eye-tracker) and conscious user preferences be reduced by appropriate parameter choice, resulting in a balanced visual message and better matching of requirements imposed on the designer?

The main target in the first part of this study was to measure the agreement of WCAG standards with user expectations of a friendly design and marketing goals associated with high visual influence on a user. We focused on the evaluation of contrast between a pictogram and backgrounds to verify different combinations from the perspective of visual intensity as a marketing goal, ranking color combinations by WCAG standards, and, in the third dimension, ranking combinations in the subjective process with input from users based on their preferences. As a result, three rankings were generated. The next step of the research was the final integrated ranking set up based on weights assigned to the three used criteria. The results show that, despite the initial asymmetry and ranking differences, the final design can be treated as a compromise between the three dimensions, and accessibility rules can be integrated within the design, together with other goals.

Finally, we propose an online tool (ColoUR Balance Tool) for the verification of color combinations, which ranks designs by integrating measurement data from subjective and objective experiments together with WCAG contrast evaluations.

## 2. Materials and Methods

In this section, the materials and methods that were used in the research are presented. The section consists of three parts: the experimental method, experimental procedure, and research procedure.

### 2.1. Experimental Method

Our approach is based on perceptual experiments. To identify color combinations that match most noticeably, we used forced-choice metrics in our experiment. Below, we explain the procedure used. 

**Figure 2:**
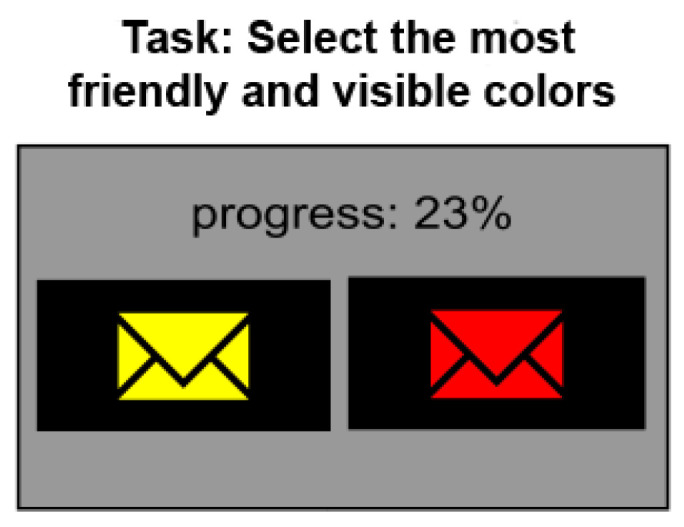
Overview of the forced-choice procedure used in the experiment. The task given to the observer was to select the most friendly and visible colors.

**The forced-choice method**. In order of the *forced-choice pairwise comparison* procedure, the observers are shown a pair of images (with the same primary color—background and different secondary colors—pictogram) and are asked to indicate the more eye-catching image (see Figure 2). Observers are always forced to choose one image, even if they see no difference between them (hence the name “forced-choice”). There is no time constraint within which one must make their decision. The method is popular, but it is highly tedious if a large number of images need to be compared. However, as reported in [23], this approach results in the smallest measurement variance and thus produces the most accurate results in comparison with other metrics used as standard in perceptual experiments, such as single stimulus, double stimulus, or similarity judgment [23].

**Choice of colors for the experiment**. Color theory encompasses a multitude of definitions, concepts, and design applications. There are three basic categories of color theory that are logical and useful: the color wheel, color harmony, and color contrast (the context of how colors are used, etc.) [24,25,26].

When choosing colors for the experiment, the following rules were considered:

- A low number of colors should be used—a maximum of 10. Too many variants could affect user perception and the course of the experiment;
- Neutral color inclusion: white, black, or gray. These colors play a calming and reassuring role in web design;
- Saturated colors are most often chosen as eye-catching elements on a website.

By analyzing the trends and design patterns for websites published on awwwards.com or colormatters.com, one can see the “Vibrant Colors Modulated by Neutral” trend. According to this, the use of basic, saturated colors as the main elements of websites is becoming more and more common, e.g., in advertisements, slogans, menus, and buttons. According to the mentioned trends, these colors are combined with neutral colors to soften them and increase visibility.

We based our choice on the traditional color wheel, also known as the Isaac Newton (1666) circular diagram, which has been the basis for a lot of research and the development of many systems and theories. In one variant, the color wheel can be divided into three parts, the primary colors: red, yellow, and blue. Mixing these colors creates secondary colors: orange, green, purple. Therefore, we based the experiment on nine colors: red, yellow, blue, orange, green, purple, black, gray, and white. 

**Figure 3:**
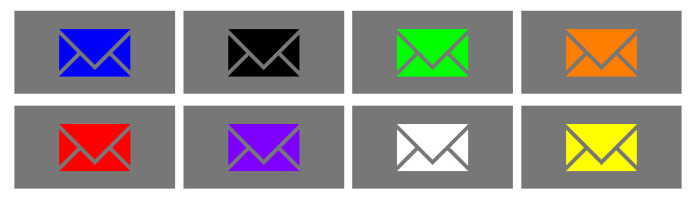
Example test images used in the experiment. In the figure, the images were composed from a “gray” color set as primary for the background, and the remaining colors (blue, grey, green, orange, red, violet, white, and yellow) were set as secondary colors.

**Characteristics of the stimulus and experiment conditions**. In order to find the most eye-catching but user-friendly pairs of colors, 72 images were prepared as follows: for each of the analyzed colors (black, gray, green, blue, violet, red, orange, yellow, and white), one color was fixed as primary (and set as a background), and the second color for each generated pictogram was chosen from the set of remaining colors and called the secondary color (and set as a pictogram). Example images with a gray primary color are depicted in Figure 3. The choice of colors for the experiment is presented in the Methods section.

**Eye-tracking metrics**. The primary requirement of eye movement analysis, in the context of a gaze-contingent system design, is the identification of fixations and saccades. Fixations naturally correspond to the desire to maintain one’s gaze on an object of interest. Saccades are considered manifestations of the desire to voluntarily change the focus of attention [27].

Henderson and Hollingworth (1998) [28] reviewed past results, indicating that the positions of fixations within a scene are nonrandom, with fixations clustering on informative scene regions. As fixations are the most common feature recorded by an eye-tracker that provides inferences about cognitive processes or states that users are interested in probing, they seemed the most suitable for our analysis. The main issue in the analysis of objective data is the identification of localization, where the user’s eyes essentially stop scanning about the scene, holding the central foveal vision in place so that the visual system can take in detailed information about what is being looked at. Several metrics can be used to evaluate the relative informativeness of scene regions, such as the total time that a region is fixated in the course of scene viewing, the first fixation duration (the duration of the initial fixation in a region), the first pass gaze duration (the sum of all fixations from first entry to first exit in a region), or the time to first fixation [27].

**Figure 4:**
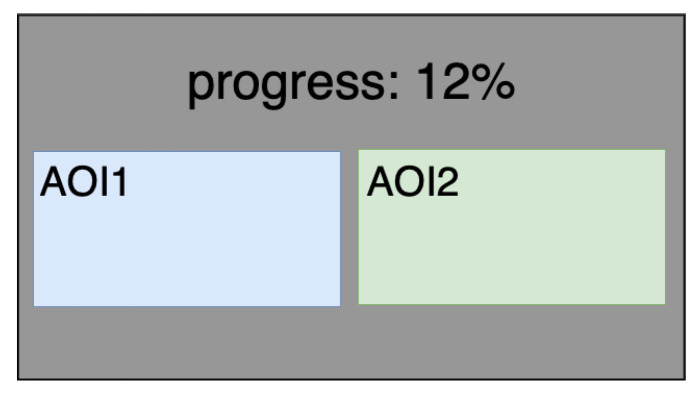
The AOIs defined to filter the ET signal for displayed images.

In our research, our focus was on the color combination that first attracts the user’s attention. As the very first impression is measured by the time to the first fixation, we used it for further analysis. This metric was measured just after the pair of images was displayed, and the image that first attracted the user’s attention was determined. To conduct the eye-tracking analysis, we defined two areas of interest, AOI1, and AOI2, for which the metric “time to first fixation” was computed (see Figure 4). Every image from the displayed pair of images was associated with one of the defined areas of interest (AOIs).

### 2.2. Experimental Procedure

The observers were asked to read written instructions before every experiment. According to rules suggested in [29], it is preferable to include at least two replications (i.e., repetitions of identical conditions) in an experiment of this type. Replications make it possible to calculate the individual reliability and, if necessary, to discard unreliable results from some subjects. In addition, replications ensure that learning effects within a test are, to some extent, balanced out. A further improvement in the handling of learning effects was obtained by including a few ‘dummy presentations’ at the beginning of each test session. The preliminary presentations were not taken into account in the statistical analysis of the test results. In our experiment, we used three ’dummy scenes’. The experiment took no longer than 30 min. Following the [29] recommendations, the experiment started with a training session in which observers familiarized themselves with the task, interface, and displayed images. To ensure that observers were fully concentrated on the experiment, three random trials were shown at the beginning of the main session in which the results were not recorded. The images were displayed in random order and with different randomization for each session. Two consecutive trials showing the same test image were avoided if possible. The experiment was performed as a whole with the use of an eye-tracker; therefore, each experiment was preceded by the calibration of the user who could only start the experiment procedure after this process. For the experiment, we used the Gazepoint GP3 HD 150 Hz eye-tracker.

The images are shown on a 50% gray background. The same background was used for the intervals between displayed pairs of images. The experiment was conducted on a Microsoft Surface Pro 4 computer with a 12-inch display that was calibrated with X-Rite i1 Publish Pro 2. For comparison, only images with the same primary color were displayed (see Figure 2). This enabled reliable comparisons by users, as introducing more than three colors (one primary and two secondaries) would make the experiment difficult to conduct or could even lead to inconsistent results.

The experiment was conducted by 41 naive observers who were confirmed to have standard or corrected-to-standard vision. The age of the observers ranged from 25 to 60 years. For additional reliability, each observer repeated the experiment three times, but no two repetitions took place on the same day in order to reduce the learning effect. We made sure that the next repetitions were not performed closer together than an interval of 2 weeks. During the three repetitions of the experiment, we collected 123 samples—41 samples per repetition. The number of participants complied with that suggested for Human–Computer Interaction (HCI) studies (at least 30 users) [30,31]. According to [23], collecting 30–60 samples per condition is also sufficient to distinguish a pair of conditions that do not differ much from each other. However, to be sure that the collected number of samples was really sufficient, we computed the effect size between the compared conditions, $u_{i}$ and $u_{j}$ using Equation (1).

(1) $$P=1-\frac{1}{\sigma \sqrt{4\pi }}\int_{-\infty }^{0}e^{\frac{-{\left(u_{i}-u_{j}\right)}^{2}}{4\sigma^{2}}}dt.$$

The *P* value was computed from the normal cumulative distribution function, assuming that the variance of the visibility difference was $2\sigma^{2}$. This probability is very useful as it estimates the percentage of cases in which the average observer will prefer one setting over another. For both preferences and ET data, the mean effect size was at the 60–70% level. Taking into account the time-cost and time-consumption of the research and the purpose of the experiment, from a practical point of view, such results seem sufficient, and we explain this further in the Discussion section.

### 2.3. Research Procedure

**Figure 5:**
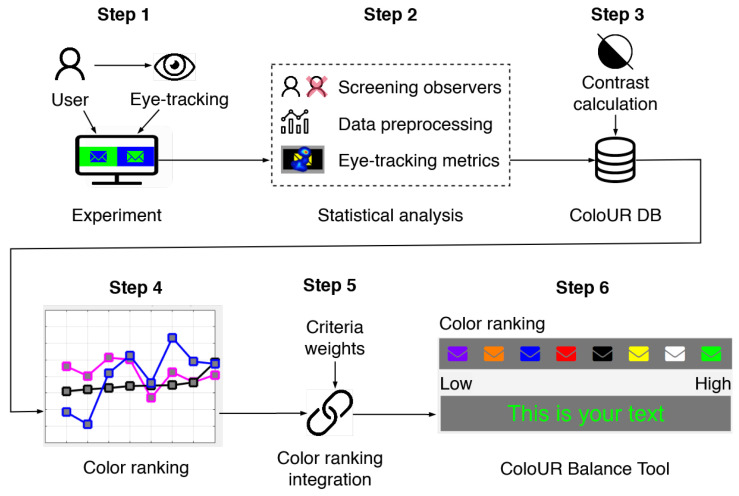
The schema shows the research procedure. The experiment with user participation allowed us to collect data on the time to first fixation for color pairs. The collected data were subjected to statistical analysis, resulting in the formation of the ColoUR DB. The ColoUR DB contains data on the natural eye fixation of users on color pairs and a contrast calculation for the same color pairs. Color ranking is based on parameters entered by the designer, i.e., criteria and their weights. The ranking can be generated for each criterion or in an integrated form. The research result is a tool for designers—the ColoUR Balance Tool.

The schema of the research procedure is depicted in Figure 5. The procedure consisted of five steps, as described below in detail.

**Step 1** was to experiment with users according to the procedure described at the beginning of this section. The experiment was carried out under controlled laboratory conditions on a group of 41 users. Users were tasked with the selection of color pairs that they perceived to be most user-friendly and allowed them to easily read messages. However, the collected and analyzed data were not only the conscious preferences of the users (subjective data), their unconscious reactions were also recorded through the eye-tracker (objective data). The users’ preferences were collected by the forced-choice procedure (see Figure 2), and the unconscious reactions were measured by a biosensor as the time to the first fixation, measured just after the pair of images was displayed. The time to first fixation metrics was computed for the image (from a pair of the images) that first caught the user’s attention via eye-sight.

**Step 2** involved statistical analysis of the data collected from the experiment: subjective and objective data. As the observers may have reported implausible impression scores because they misunderstood the experiment instructions or did not engage in the task and gave random answers, observer screening was employed. To be able to compare results obtained for individual color pairs, data were standardized and normalized to the whole space.

**Screening Observers**. The observers may have reported implausible impression scores because they misunderstood the experiment instructions or did not engage in the task and gave random answers. If the number of participants is low, it is easy to spot unreliable observers by inspecting the plots, so a screening procedure should be employed. We used the approach described in [29] standard, (Annex 2.3.1), which is a numerical screening procedure. The procedure involves counting the number of trials in which an observer’s result lies outside the ±2 standard deviation range and rejecting those observers for whom (a) more than 5% of the trials are outside that range and (b) the trials outside that range are evenly distributed so that the absolute difference between the number of trials exceeding the lower and upper bounds of that range is not more than 30%. We performed this procedure on our data and found that four participants needed to be removed.

All analyzed data were fully anonymized. Before the experiment, the participants provided informed written consent for data from the perceptual experiment to be used in our research (according to the Bioethics Committee Agreement no KB-0012/24/2020).

**Data preprocessing**. To stabilize the results between images given for the same primary color, the results were standardized, but this was done separately for every primary color. Different people are likely to use different evaluation methods when rating images, resulting in a different scale being associated with each observer. The easiest way to unify the scales across observers, and thus make their data comparable, was to apply a linear transformation to equalize the means and the standard deviations for all observers in the form of a z-score (see Equation (2)).

(2) $$z_{i,j}=\frac{d_{i,j}-\bar{d_{i}}}{\sigma_{i}}$$

where the mean $\bar{d_{i}}$ and standard deviation $\sigma_{i}$ were computed as usual across all images for a given primary color by an *i*-th observer; *j* is the index of the secondary color in the image: the higher the value of the metrics, the more eye-catching the image is.

**Figure 6:**
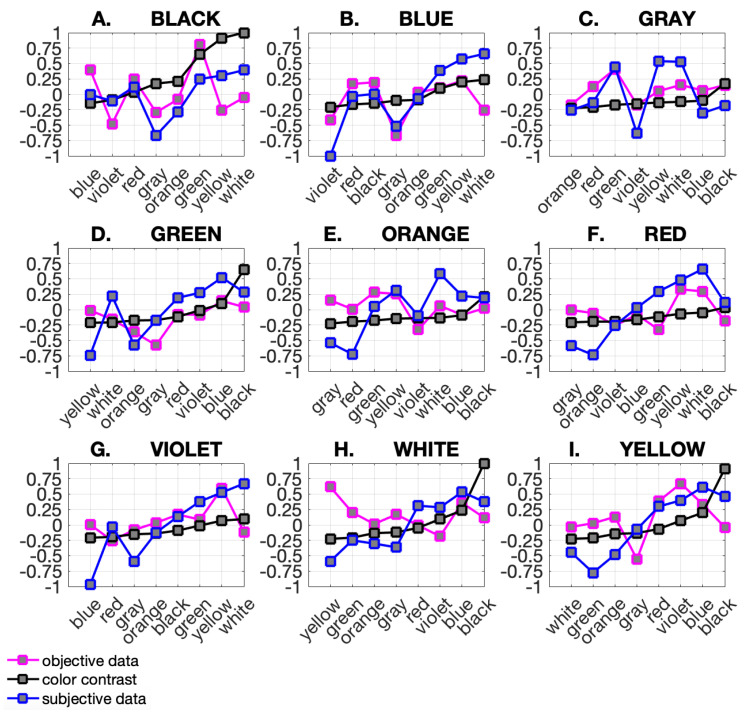
Color rankings for primary and secondary colors were developed based on ColoUR DB. The figure depicts the results for 9 tested colors: (**A**–**I**). The color ranking is arranged according to the level of user eye attraction. Each graph only applies to a single primary color, for example, (**A**)—Primary color Black. On the X axis, there is a set of secondary colors. The Y axis represents normalized values obtained in the experiment. Each graph shows tree plots. The first one (magenta) shows objective data recorded by an eye-tracker. The second one (black) shows a contrast calculation for the same color pairs. The contrast was determined according to the WCAG standard presented in the Methods section. The third one (blue) shows subjective data coming from users’ responses obtained during the experiment, as presented in the Results section.

After standardization, data were normalized, which enabled easier comparison of the results. The results for every primary color are depicted in Figure 6. Every plot contains the color ranking arranged according to the contrast of secondary colors.

During the experiment, users evaluated nine primary colors: black, gray, green, blue, violet, red, orange, yellow, and white. Each primary color was fixed as a background, and the remaining colors were set as secondary colors, i.e., foreground—pictogram (see Figure 3). The goal was to investigate whether a change in the primary color influenced user assessment of user-friendliness and visibility (subjective data). Together with the collection of user preferences, the user’s eye movements were recorded by the eye-tracker (objective data). The aim was to investigate, as above, whether a change in the primary color influenced user attention attachment and to measure the difference between subjective and objective user responses.

**Figure 7:**
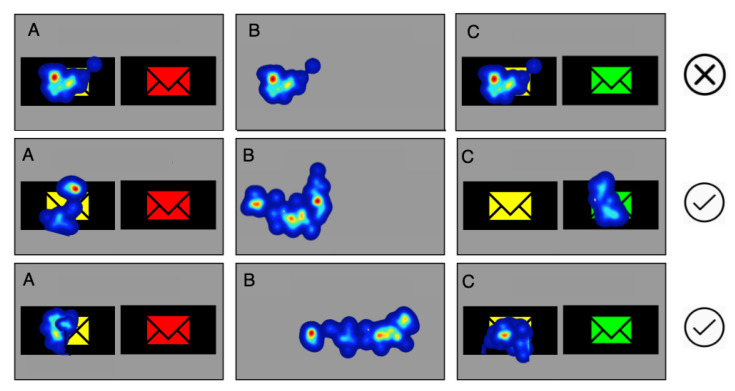
Attracting eyesight to the displayed stimulus. (**A**)—first slide, (**B**)—empty (middle-gray) screen (2 s), (**C**)—next slide. **Top**: situation where the eyes remain on the picture following the previous task. **Middle** and **Bottom**: eyes naturally resting on the image after the stimulus is displayed. To avoid situations where the user’s sight remains on the stimulus of the previously performed task and changes are noticed between images displayed one after another, an empty gray screen was shown for two seconds.

**Eye-tracking metrics calculation**. Depending on the situation, the time to the first fixation was determined in different ways: if the fixation resulted from keeping eyesight on the previous slide (see Figure 7 (Top)), then such a situation was not taken into account. In the calculations, we used only situations where fixation began after a pair of images was displayed. The procedure for these situations is depicted in Figure 7 (Middle), where the gaze remained on the place where the subject’s eyes had focused for a while, but after the picture was displayed, it automatically moved to the place that attracted attention, and in Figure 7 (Bottom), where the gaze moved freely around the experiment window, but when the image was displayed, the gaze automatically moved to the image that caught the attention, even if it was the same area that caught the attention last on the previous slide. On average, 75% of cases per user met the criterion of fixation starting after the image was displayed, which corresponded to the natural situation of attracting the user’s sight to the displayed stimulus. To avoid situations where the user’s sight remained on the stimulus of the previously performed task and changes between images displayed one after another was noticed, an empty gray screen was shown for two seconds.

It is worth emphasizing that the user task was performed to determine the color combination which attracted the participants’ attention in the most friendly and visible way (Paragraph: Experimental method). The unconscious attraction of the eye to the stimulus as the very first reaction was often independent of the user’s conscious choice, which is indicative of the actual strength of the stimulus that attracted the user’s attention.

**Step 3** involved the collection of all data from the conducted experiment and the creation of a new database, named ColoUR DB, which contains the links between colors on subjective and objective levels. The ColoUR DB also includes data on the level of contrast for each of the tested pairs of primary and secondary colors. The contrast was calculated according to the formula recommended by the WCAG standard.

**Contrast calculation**. In general, contrast is connected to visual system sensitivity because of the differences between viewed stimuli [32]. There are many possible definitions for contrast, wherein some include color, and others do not. Such a multiplicity of contrast notions is extremely inconvenient; various definitions of contrast are used in different situations. The most famous contrast definitions are known as the Weber contrast [33], Michelson contrast [34], and RMS contrast [35]. In this article, we needed a formula for the level of contrast between two colors. The contrast between the background and the foreground was investigated in the visual communication area of a website. Therefore, we used the color contrast formula recommended by the WCAG standard [8]. According to these suggestions, an appropriate contrast between text and background eases and, for some users, allows the reading of information contained on websites.

WCAG standard 2.1 distinguishes two success criteria that define the required contrast levels for website elements in Guideline 1.4:

- Minimum contrast (AA level): Contrast between the background and foreground of at least 4.5:1 (or at least 3:1 for big font size);
- Increased contrast (AAA level): Contrast between the background and foreground of at least 7:1 (or at least 4.5:1 for big font size).

A large font size means at least 18 points or, in the case of bold text, 14 points. A contrast coefficient of 1:1 is the worst contrast—white text on a white background (the best contrast is black text on a white background)—and its coefficient is 21:1.

The WCAG standard defines contrast using a numerical approach as a ratio between the luminance (brightness) components of two colors, calculated based on their RGB components. The color contrast was calculated using the procedure recommended in WCAG standard:

(i) Measure the relative luminance of each letter (unless they are all uniform) using the formula L=0.2126·R+0.7152·G+0.0722·B where R, G and B are defined as: if $R_{sRGB}\leq 0.03928$ then $R=R_{sRGB}÷12.92$ else $R={\left(\left(R_{sRGB}+0.055\right)÷1.055\right)}^{2.4}$, if $G_{sRGB}\leq 0.03928$ then $G=G_{sRGB}÷12.92$ else $G={\left(\left(G_{sRGB}+0.055\right)÷1.055\right)}^{2.4}$, if $B_{sRGB}\leq 0.03928$ then B=B+sRGB÷12.92 else $B={\left(\left(B_{sRGB}+0.055\right)÷1.055\right)}^{2.4}$, and $R_{sRGB}$, $G_{sRGB}$, and $B_{sRGB}$ are defined as: $R_{sRGB}=R_{8bit}÷255$, $G_{sRGB}=G_{8bit}÷255$, $B_{sRGB}=B_{8bit}÷255$.
(ii) Measure the relative luminance of background pixels immediately next to the letter using the same formula.
(iii) Calculate the contrast ratio using the following formula: (L1+0.05)÷(L2+0.05) where L1 is the relative luminance of the lighter of the foreground or background colors, and L2 is the relative luminance of the darker of the foreground or background colors.
(iv) Check that the contrast ratio is equal to or greater than 7:1 It is also possible to use the Color Contrast Analyser software recommended by the WCAG (https://www.tpgi.com/color-contrast-checker accessed on 1 September 2021).

To be able to compare results obtained for individual color pairs, data were standardized and normalized to the whole space.

**In Step 4**, a color ranking was generated for each criterion, i.e., subjective preference, objective measurements, and color contrast based on WCAG. The ranking was generated for each primary and secondary color pair and ordered from the least matching criteria to the best matching (low to high). The rank for each primary color is shown in Figure 6. Primary colors are marked with letters A to I. On the X-axis, there is a set of secondary colors. The Y-axis represents standardized and normalized values obtained in the experiment. The ranking is color-coded: magenta for objective data recorded by an eye-tracker, blue for subjective data coming from users’ responses obtained during the experiment, and black for color contrast.

**Step 5** involved a method of criteria integration to obtain a single color ranking. Each criterion was assigned a weight. Those weights determined the importance level of each criterion, e.g., the size of the influence that the WCAG standard should have on the ranking. Weights can be set to one of 4 levels. Level 0 means that the criterion is disabled and does not participate in a color ranking generation. Level 1 is the lowest criterion weight (for subjective and objective data) or may indicate that the WCAG standard has not been fulfilled (for color contrast). Level 2 represents an average criterion weight (for subjective and objective data) or shows that the WCAG standard has been fulfilled on the AA level (for color contrast). Level 3 represents the highest criterion weight (for subjective and objective data) or shows that the WCAG standard has been fulfilled at the AAA level (for color contrast). Choosing criteria and assigning weights is the designer’s task. Depending on the visual message type, the designer can choose which characteristics are most important to the project.

**Color ranking integration and criteria weights.** The color ranking is generated based on parameters entered by the designer. Those parameters are assigned using three criteria: color contrast, subjective, and objective data. For each criterion, the designer sets weights that indicate the importance of these criteria when ranking colors. Color ranking integration (CRI) is calculated using Equation (3):

(3) $$CRI=\left(a\cdot w_{a}\right)+\left(b\cdot w_{b}\right)+\left(c\cdot w_{c}\right)$$

where *a* is the color contrast criterion, *b* is the subjective data criterion, *c* is the objective data criterion, and $w_{x}$, x={a,b,c} are criteria weights. Criteria weights can be set to one of four levels, and $w_{x}=\left\{0,1,2,3\right\}$. For the color contrast criterion, $w_{a}$ has the following categories: 0=null means that the criterion is disabled and does not participate in color ranking generation; 1=rejected means that the WCAG standard has not been fulfilled; 2=acceptedAA means that the WCAG standard is fulfilled at the AA level; and 3=acceptedAAA means that the WCAG standard is fulfilled at the AAA level.

For subjective data $w_{b}$ and objective data $w_{c}$ criteria, the following categories were used: 0=null means that the criterion is disabled and does not participate in the color ranking generation; 1=low is the lowest criterion weight; 2=medium indicates the average criterion weight; and 3=high is the highest criterion weight. Modification of criteria choices and weights causes changes in the color ranking. The choice must be well thought out, taking visual message application and goals into account.

**In Step 6**, we used the results to develop and implement a tool for designers that we call The ColoUR Balance Tool. The ColoUR Balance Tool retrieves data from the ColoUR DB acquired earlier during the perceptual experiment. The ColoUR DB contains 72 combinations of primary and secondary colors. The ColoUR Balance Tool is presented in the Results section with a case study.

## 3. Results

The following section discusses the results of a perceptual experiment with the main goal of analyzing different color combination (two colors) rankings: subjective (according to users’ preferences), objective (attracts the users’ attention, measured by an eye-tracker), and WCAG contrast as the standard procedure. We aimed to identify color connections that are attractive from the perspectives of commercial goals and user experience, as well as those that are compliant with the WCAG standard.

**Figure 8:**
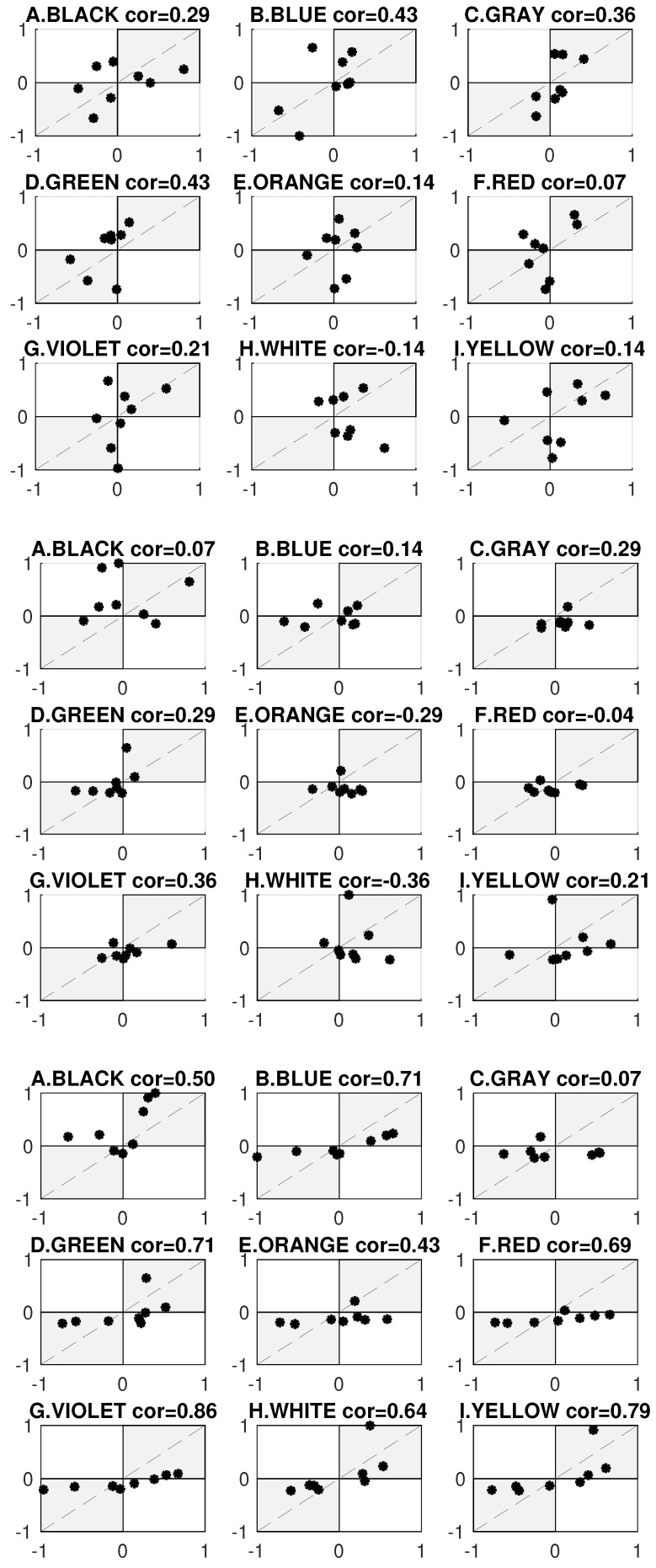
Kendall correlation between (**Top**) the subjective data received from the experiment in the form of user preferences and the objective data represented by time to the first fixation recorded by the eye-tracker; (**Middle**) the objective data and WCAG contrast; (**Bottom**) the subjective data received from the experiment in the form of user preferences and WCAG contrast.

The color rankings shown in Figure 6 represent the main result of the performed research. The color ranking is ordered by contrast values. Results are presented for primary colors A through I and linked to a set of secondary colors (X-axis). All data are standardized and normalized. Discrete values are connected by a line for better data visualization. The results indicate a high level of asymmetry in human behavior. Subjective and objective data provided from the same user were found to differ dramatically. To check the dependence between the data, the Kendall correlation was computed. The Kendall correlation results are depicted in Figure 8 (Top) and in Table 1, where the correlation value cor and significance level *p* are recorded. The mean Kendall correlation was equal to 0.2460, and the statistical significance was not lower than the threshold of 0.05 for any of the primary colors. This means that the data are quite different. The results show the asymmetry as well the visibility of objective data and contrast values, as shown in Figure 8 (Middle). Here, similar to above, the mean Kendall correlation was low and equal to 0.2263, and the significance level is above the 0.05 threshold. This means that WCAG standards do not correspond to the natural eye-catching mechanism. The biggest similarity was between the subjective data and contrast (see Figure 8 (Bottom)). Here, the mean Kendall correlation was equal to 0.60. This value is not particularly impressive, but for 6 of the 9 primary colors (blue, green, red, violet, white, and yellow), the Kendall correlation significance level was lower than 0.03. This means that the influence of the WCAG standard on visibility is consistent with user preferences.

**Figure 9:**
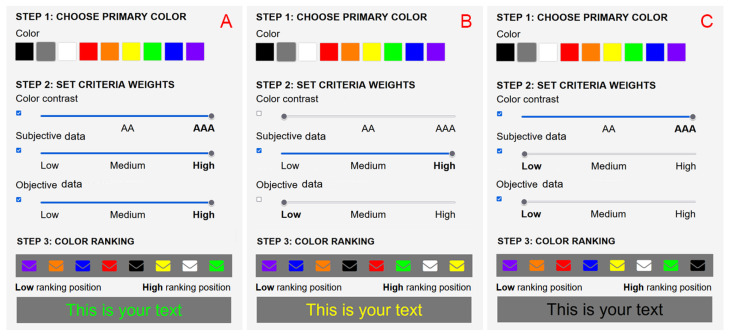
The research results were made available with the ColoUR Balance Tool.

**ColoUR Balance Tool and ColoUR DB**. The research results were implemented as a tool for designers—the ColoUR Balance Tool, an application that runs in a user interface design. The ColoUR Balance Tool allows a primary color to be elected and the remaining colors visualized as pictograms or text. Figure 9 shows example screens and the sample selection process. The results are displayed to the designer in the form of a color ranking, the time to the first fixation, and user-friendliness according to users’ votes. The ColoUR Balance Tool retrieves data from ColoUR DB acquired earlier during the perceptual experiment. The ColoUR DB contains 72 combinations of primary and secondary colors. There are two metrics for each color pair: the friendliness calculation and the time to the first fixation determined by user natural behavior. An additional metric is color contrast, which is calculated mathematically for each color pair according to the WCAG standard. Ultimately, the ColoUR Balance Tool presents a color ranking, which is calculated based on parameters set by the designer. The ranking is displayed on the scale from lowest to highest, where the lowest means the color pair that least matches the parameters defined by the designer.

Tool usage is based on three steps (see Figure 9). In the first step, one of nine primary colors is chosen. This will be the visual message’s background. In the second step, criteria and their weights that are relevant to visual message design can be chosen: color contrast, subjective data, and objective data. Each of the criteria has 3 possible weights and the possibility of being disabled. Color contrast does not fulfill the standard, AA, and AAA, which is consistent with the WCAG standard levels. Subjective data and objective data criteria can be set to low, medium, and high levels. In the third step, a color ranking is presented where “low” describes the color pair that fulfills the criteria the least and “high” describes the pair that matches the criteria the most. A graphical representation of the research results consists of pictograms and text. A case study follows:

**Case A** In this case study, gray was chosen as the primary color. In the second step, all criteria were enabled, and all weights were set to their maximum values, i.e., color contrast—AAA, subjective data—high, objective data—high. The color ranking shows how the gray color matched the other colors. Color matches rank from lowest to highest in terms of criteria settings and their weights. In this case, the gray color matched best with green and then white and yellow.
**Case B** In this case study, we kept gray as the primary color. Criteria and their weights were modified. The color contrast criterion and objective data were disabled and did not influence the color ranking. Only subjective data were left and were given the highest weight—high. In this configuration, the color ranking changed. The gray color matched best with yellow and then white and green.
**Case C** In this case, all criteria were enabled but with different weights. The color contrast criterion was set to the highest weight—“AAA” and subjective data and objective data had the lowest weight—“low”. In this configuration, gray matched best with black and then green and white.

The ColoUR Balance Tool is available on Github: https://visual-communication.github.io/ColoUR-Balance (accseed on 29 September 2021). In the GitHub repository is the HTML code with data from the experiment and the method used to calculate the ranking and link to the Color Balance Tool. The repository is available on an MIT license.

## 4. Discussion

The main task carried out in the paper was an attempt to find convergence between asymmetric user behavior measured objectively by an eye-tracker and subjectively by collecting user preferences together with accessibility rules. The experiment involved 41 naive observers who were confirmed to have standard or corrected-to-standard vision. This resulted in the collection of 41 samples from each from three repetitions. The number of participants complies with that suggested for Human–Computer Interaction (HCI) studies (at least 30 users) [30,31]. According to [23], collecting 30–60 samples per condition (two matched colors represented by a pictogram and background) is also sufficient. For both preferences and ET data, the mean effect size was 60–70% which, from a practical point of view, seems sufficient. As reported in other papers [35,36], research results should be considered in connection to their domain. Statistical significance does not mean practical significance. Only by considering the context can one determine whether a difference is practically significant (that is, whether it requires action). This individual approach to research results is often used in marketing, user experience, and service design [37]. It means that the error value found during the medical data analysis has a completely different influence on system behavior in comparison with the assessment of the tendency in the visual message. In the latter case, all tendencies and suggestions that increase the user experience and visual design quality of the multimedia message are important.

A group of people aged from 25 to 60 participated in this study. The Bioethical Commission issued research permission for those aged above 18 years old. Perceptive research is most commonly carried out on adults. The use of a higher age limit was connected with human sight characteristics in the color perception domain. Research shows that, with age, color perception disorders build up [38] and contrast sensitiveness emerges [39]. They most frequently build-up at ages of 60–65 and older. The causes for color disorders are changes that occur in the eye lens and retina surface. Decreases in the numbers of stamens and suppositories and the quality of their functioning worsen color recognition [40]. Contrast sensitivity is connected with a decrease in the amount of light that reaches the retina, which leads to an increase in internal light diffusion and aberration. This causes a lowering in the contrast of perceived image [41]. For these reasons, our research was limited to those below 60 years of age.

The conducted research allowed us to identify the dominant colors that meet the requirements of commercial goals focused on visual intensity, user preferences, and WCAG standards dedicated to web design. According to subjective preferences, the best-rated colors were secondary yellow, green, and white. They dominated in the primary groups A—Black, B—Blue, C—Gray, F—Red, and G—Violet. The mentioned colors induced positive feelings among users. It is noteworthy that the symbolism of such colors is quite universal in many cultures: yellow—joy, optimism, creativity; green—health, youth, nature; white—purity, innocence, a new beginning [42]. The combination of these colors with background colors (gray or black) is harmonic for the recipient and at the same time legible and visible.

In the analysis of the users’ reactions by eye-tracking (objective data), users’ attention was found to be drawn, as for user preferences, to yellow and green secondary colors, but also to black, red, and blue. They dominate in the primary groups: A—Black, B—Blue, I—Yellow. Objective measurements are connected with commercial goals and attempt to impact users’ behaviors through intensive visual content, and this is not always pleasant from the perspective of users’ targets and user experience. Objective data given by eye movements and gaze points recorded by eye-tracking, subjective preference, and color contrast indicated high asymmetry in users’ reactions. The subjective preferences of the users did not concur with the reaction of the eyes to the same colors. Within one analyzed primary color, a different group of colors evoked friendliness than those that attracted the user’s attention. An example is group A—Black where, according to users’ preferences, the most friendly connection is Black-White, but in the eye-tracking study, attention was drawn to Black-Green (white shifted 5 positions lower).

An interesting observation is the fact that there is some kind of compliance between subjective user preferences and the WCAG standard for designing websites. This means that the use of WCAG standard rules is beneficial not only for people with disabilities but for all users which, as confirmed in earlier studies [12].

Our results, obtained from objective measurements confirm the results reported in [3,43], showing that bright, saturated colors attract user attention most strongly. Too much color in a large area overstimulates the retinas which can strain the eyes. This could be irritating for a user. However, the use of such techniques for a short period can effectively attract the user’s attention.

Moreover in [3,43], it was concluded that bright and saturation color properties are more important for drawing attention than hue, and two other studies [44,45] found that bright, saturated colors have links to high arousal. This brings us to the conclusion that eye-catching colors are characterized by having high hue and saturation values, while the preferred and user-friendly colors combination are based more on contrast.

**Research application:** The research has applications in broadly understood websites and the design of other multimedia components. Designs delivered by the proposed tool can be scaled for different target groups, and their priorities can be defined by assigned weights. Instead of creating separate designs, a single version can be proposed. It can also be used within marketing platforms where typical target groups are defined by demographic characteristics but not visual abilities.

The research gives the designer the ability to better match their visual message with the project’s requirements. Advertisements and commercial messages can be better matched through color contrast criteria and objective measurements. Informative visual messages where aesthetics and color friendliness plays important roles can be better matched through subjective preference criteria. Additionally, by applying color contrast, visibility can be increased, e.g., for visually impaired and older people.

The practical result of the presented research is the development of the ColoUR Balance Tool https://visual-communication.github.io/ColoUR-Balance. This could become one of the tools used in a designer’s work in addition to solutions such as Paletton (paletton.com), Colourlovers (colourlovers.com), and ColorAdobe (color.adobe.com) and the configuration of visual messages within advertising servers or planning-conversion-focused experiments within dedicated platforms.

## Figures and Tables

**Table 1:** Kendall correlations and significance level results for objective data (ET), subjective data (P), and contrast (C). Values with p<0.5 are marked in bold and underlined.

*Primary*	ET/P		ET/C		P/C	
*Color*	corr	*p*	corr	*p*	corr	*p*
**black**	0.2857	0.3988	0.0714	0.9049	0.5000	0.1087
**blue**	0.4286	0.1789	0.1429	0.7195	**0.7143**	**0.0141**
**gray**	0.3571	0.2751	0.2857	0.3988	0.0714	0.9049
**green**	0.4286	0.1789	0.2857	0.3988	**0.7143**	**0.0141**
**orange**	0.1429	0.7195	−0.2857	0.3988	0.4286	0.1789
**red**	0.0714	0.9049	−0.0364	1.0000	**0.6910**	**0.0200**
**violet**	0.2143	0.5484	0.3571	0.2751	**0.8571**	**0.0017**
**white**	−0.1429	0.7195	−0.3571	0.2751	**0.6429**	**0.0312**
**yellow**	0.1429	0.7195	0.2143	0.5484	**0.7857**	**0.0055**
**mean**	**0.2460**	**0.5159**	**0.2263**	**0.5466**	**0.6006**	**0.1421**

## Data Availability

Data are available in the GitHub repository at https://visual-communication.github.io/Colour-Balance. There is HTML code with data from the experiment and the method used to calculate the ranking as well as a link to the Color Balance Tool. The repository is on an MIT license.
